# The impact of intermittent fasting on gut microbiota: a systematic review of human studies

**DOI:** 10.3389/fnut.2024.1342787

**Published:** 2024-02-12

**Authors:** Isa Paukkonen, Elli-Noora Törrönen, Johnson Lok, Ursula Schwab, Hani El-Nezami

**Affiliations:** ^1^Institute of Public Health and Clinical Nutrition, School of Medicine, University of Eastern Finland, Kuopio, Finland; ^2^Department of Medicine, Endocrinology and Clinical Nutrition, Kuopio University Hospital, Kuopio, Finland; ^3^Molecular and Cell Biology Research Area, School of Biological Sciences, The University of Hong Kong, Pokfulam, Hong Kong SAR, China

**Keywords:** gut microbiota, intermittent fasting, time-restricted eating, alternate day fasting, 5:2 diet, Ramadan fasting, human studies, systematic review

## Abstract

**Background:**

Intermittent fasting (IF) has gained popularity in interventions targeting overweight, obesity and metabolic syndrome. IF may affect the gut microbiome composition and therefore have various effects on gut microbiome mediated functions in humans. Research on the effects of IF on human gut microbiome is limited. Therefore, the objective of this systematic review was to determine how different types of IF affect the human gut microbiome.

**Methods:**

A literature search was conducted for studies investigating the association of different types of IF and gut microbiota richness, alpha and beta diversity, and composition in human subjects. Databases included Cochrane Library (RRID:SCR_013000), PubMed (RRID:SCR_004846), Scopus (RRID:SCR_022559) and Web of Science (RRID:SCR_022706). A total of 1,332 studies were retrieved, of which 940 remained after removing duplicates. Ultimately, a total of 8 studies were included in the review. The included studies were randomized controlled trials, quasi-experimental studies and pilot studies implementing an IF intervention (time-restricted eating, alternate day fasting or 5:2 diet) in healthy subjects or subjects with any disease.

**Results:**

Most studies found an association between IF and gut microbiota richness, diversity and compositional changes. There was heterogeneity in the results, and bacteria which were found to be statistically significantly affected by IF varied widely depending on the study.

**Conclusion:**

The findings in this systematic review suggest that IF influences gut microbiota. It seems possible that IF can improve richness and alpha diversity. Due to the substantial heterogeneity of the results, more research is required to validate these findings and clarify whether the compositional changes might be beneficial to human health.

**Systematic Review Registration:**

https://www.crd.york.ac.uk/prospero/, identifier CRD42021241619.

## 1 Introduction

Fasting means the abstinence from consuming food and/or beverages for different periods of time (1). It is different from calorie restriction (CR) which can be defined as reducing daily calorie intake over a given period without malnutrition (2, 3). One way to classify fasting is dividing it into intermittent fasting (IF) and prolonged fasting (PF) (4). In the latter, only water but no food is consumed for two or more consecutive days (5). In this systematic review, the sole focus of interest was IF.

IF interventions have been used in treating several diseases, such as obesity and metabolic syndrome, and it has been suggested that the positive effects may at least be in part mediated by the gut microbiota (6–8). IF has also gained popularity as a form of lifestyle and has long roots of existing in religious and cultural contexts (9). IF can be divided into different subtypes depending on the duration of the fasting period (10). Most common forms include time-restricted eating (TRF) where the fasting period occurs within 24 h and the length varies between 12-18 h a day. An example of TRF is Islamic religion associated Ramadan fasting. Other forms of IF include alternate day fasting (ADF) which involves alternating days between *ad libitum* eating days and fasting days. A 5:2 diet is a modified version of ADF where fasting takes place in determined 2 days during the week and 5 days left are *ad libitum* eating days. During the fasting period of TRF, the number of calories consumed is typically as close to zero as possible, zero-calorie drinks such as water, black coffee and tea can be consumed (11). However, it is important to note that TRF does not necessarily result in a decrease in the overall daily calorie intake (3, 12). During ADF fasting days, the number of calories allowed to be consumed is typically around 25% of the energy requirement (11).

In previous studies with human subjects, IF has been seen to have positive effects on weight loss, composition of adipose tissue, blood pressure, anti-inflammatory processes, and autoimmune function (13–16). Some of the mechanisms by which IF may improve metabolic health include reduced free radical production, improved glucose homeostasis, augmented stress resistance and suppressed inflammation. These may be at least partially mediated by the gut microbiota as it has been found to serve a role in glucose metabolism and inflammation through microbial metabolites (17, 18). The gut microbiota and its associations for metabolic health is therefore an important target to be further investigated.

Currently only limited data exist on how IF affects the gut microbiota in humans. Consequently, the overall aim of this systematic review was to review the available literature on how different types of IF interventions affect gut microbiota richness, alpha and beta diversity, and composition in human subjects.

## 2 Materials and methods

This systematic review was registered with PROSPERO (RRID:SCR_019061). Guidelines for the Preferred Reporting Items for Systematic Reviews and Meta-Analyses PRISMA2020 (RRID:SCR_021053) checklist was used for structuring the review (19).

### 2.1 Search strategy

The first literature search was conducted on January 15, 2021 in the following databases: Cochrane Library (RRID:SCR_013000), PubMed (RRID:SCR_004846), Scopus (RRID:SCR_022559) and Web of Science (RRID:SCR_022706). Studies published from the inception of each database until January 15, 2021 were taken into account. The search was limited to human studies published in English. The second literature search was performed on June 4, 2021 in the same four databases, using the same search phrases and limitations, but this time additionally limiting the search to year 2021 in order to target potential new publications. The publication by Ozkul et al. (20) was obtained directly from the authors upon request via ResearchGate (RRID:SCR_006505).

### 2.2 Eligibility criteria

At first, only studies with TRF as the intervention were included, but the inclusion criteria were later expanded to include all forms of IF (TRF, ADF, the 5:2 diet) due to the small number of existing TRF studies.

The following study/publication types were excluded: pre-clinical and animal, observational studies, protocols, reviews, editorials, opinion pieces and case reports. Randomized controlled trials (RCTs), quasi-experimental studies and pilot studies were included.

Only studies with human subjects were included, with no further restrictions regarding participant age, sex, ethnicity *et cetera*. Studies conducted on both healthy subjects and subjects with any disease were all included.

Studies examining at least one of the following outcomes were included: gut microbiota richness, alpha and beta diversity, and composition.

### 2.3 Selection process

In the first literature search conducted on January 15, 2021, the combined result of the four databases was a total of 1,172 records. After removing the duplicates using EndNote (RRID:SCR_014001), the number of records was 831. Three reviewers (Isa Paukkonen, Elli-Noora Törrönen, Heikki-Mikael Smolander) screened the 831 records independently by titles and abstracts using Rayyan QCRI (RRID:SCR_017584) (21). If eligibility was unclear, the full text article was obtained and reviewed. After the screening, the reviewers were unblinded and the results of the screening process were discussed. Any disagreement was resolved via discussion until consensus was reached.

Based on the initial screening of titles and abstracts, 22 articles were selected, and the full texts of these articles were obtained for screening. The three reviewers (IP, ET, HS) first screened the full text articles independently, and after unblinding, reached consensus via discussion. Ultimately, four papers meeting the eligibility criteria were included. At this stage, the intervention was limited to TRF only. The excluded studies did not fit the definition of TRF and investigated other types of fasting and CR interventions, such as very-low calorie diet (VLCD) and Buchinger fasting.

However, four studies were considered to be limited for a proper systematic review, and thus the inclusion criteria were expanded to include all different forms of IF: TRF, ADF and the 5:2 diet. Of the 22 full text articles selected on the first round of screening, two new papers were included. After this, the 831 records were independently screened by titles and abstracts on Rayyan QCRI (RRID:SCR_017584) for the second time with the expanded eligibility criteria. Unfortunately, this resulted in no new papers.

To find more papers, a second literature search was performed on June 4, 2021 using the same four databases and search phrases and limitations, but limiting the search to year 2021 to target potential new publications. This search resulted in a total of 160 records of which 109 remained after removing duplicates with EndNote (RRID:SCR_014001).

After independently screening the records by titles and abstracts on Rayyan QCRI (RRID:SCR_017584), one of the three reviewers (HS) withdrew from the project. The remaining two reviewers (IP, ET) were unblinded and discussed the results of the screening process. A consensus was reached, and two studies were selected based on the titles and abstracts. The full text of these articles was obtained and screened independently. After unblinding and discussion, both papers were concluded to meet the eligibility criteria.

All in all, the two literature searches yielded 1,332 results, of which 940 remained after removing duplicates. In the end, a total of eight studies were included in the review: six TRF studies, one ADF study and one 5:2 diet study.

### 2.4 Data collection process and items

The eight papers were divided evenly between the two review authors (IP, ET), who independently collected data from the papers, but worked closely together on the manuscript and discussed the findings thorough the whole writing process.

From each included study, data were sought for the following outcomes: gut microbiota richness, alpha and beta diversity, and composition. All results that were compatible with each outcome domain in each study were sought (all measures, time points, analyses). Data were also sought for the following variables: study and participant characteristics, weight and body mass index (BMI), and dietary information. Making any assumptions regarding missing or unclear information was avoided; in case some original information was missing or unclear, this is stated in the review.

### 2.5 Risk of bias assessment

For risk of bias assessment, Cochrane’s RoB 2 tool (22) was used for RCTs and Cochrane’s ROBINS-I tool (23) for the non-randomized studies, as recommended by Arnesen et al. (24). The non-randomized studies included both uncontrolled, one group studies and non-randomized controlled trials, i.e., the study had intervention and control groups, but the group allocation was not random. Two reviewers (IP, ET) rated the bias independently, and after unblinding, reached consensus via discussion.

## 3 Results

### 3.1 Study selection and characteristics

**Figure 1:**
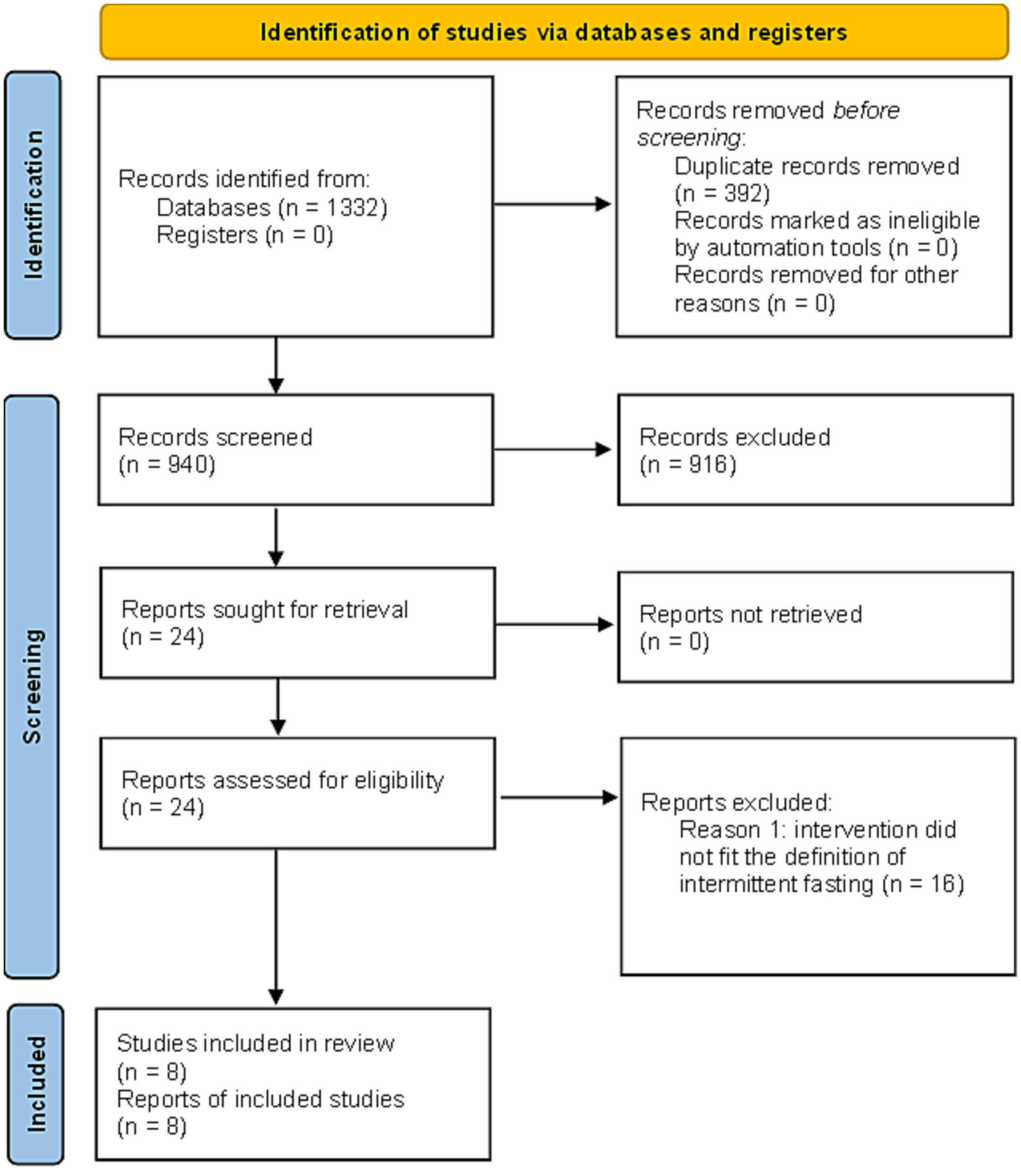
PRISMA2020 (RRID:SCR_021053) flow diagram for new systematic reviews which included searches of databases and registers only (19). The results from the first and second literature search are combined. Duplicate records were removed with EndNote (RRID:SCR_014001).

The results of the search and selection process are presented in Figure 1 using a PRISMA2020 (RRID:SCR_021053) flow diagram for new systematic reviews which included searches of databases and registers only (19).

Citation of each included study and the study and participant characteristics are presented in Table 1.

**Table 1:** Study and participant characteristics.

Study citation (First author, title, year of publication)	Country	Study design	Sample size (M/F)	Age (years) (expressed as mean ± SD unless stated otherwise)	Participant characteristics	Description of intervention	Description of control	Intervention duration
Guo et al. Intermittent fasting improves cardiometabolic risk factors and alters gut microbiota in metabolic syndrome patients, 2021.	China	RCT	46 M/F distribution was only reported for those who completed the intervention. total: 39 (21/18) 5:2 diet: 21 (10/11) control: 18 (11/7)	Inclusion criteria: 30-50 years Mean ± SD age was only reported for those that completed the intervention. 5:2 diet: 40.2 ± 5.7 control: 42.7 ± 4.1	Metabolic syndrome patients	2-day IF (5:2 diet) which involved a 75% of energy restriction for 2 non-consecutive days a week and an *ad libitum* diet the other 5 days.	An *ad libitum* diet	8 weeks
Cignarella et al., Intermittent fasting confers protection in CNS autoimmunity by altering the gut microbiota. 2018.	United States	RCT (pilot trial)	17 M/F distribution was only reported for those who completed the intervention. total: 16 (4/12) ADF: 8 (3/5) control: 8 (1/7)	Inclusion criteria: 18-60 years Mean ± SD age was only reported for those that completed the intervention. ADF: 40 ± 12 control: 42 ± 8.2	Relapsing multiple sclerosis patients Inclusion criteria: BMI 23 kg/m^2^ or higher	ADF for 15 days plus the same steroid regimen as the control group. Fasting days calories limited to 500 kcal per day.	An *ad libitum* control group in which patients received corticosteroid treatment.	15 days
Zeb et al. Effect of time-restricted feeding on metabolic risk and circadian rhythm associated with gut microbiome in healthy males, 2020.	China	RCT	total: 80 (80/0) TRF: 56 (56/0) control: 24 (24/0)	Inclusion criteria: young aged Participant age range or mean ± SD age not stated	Young healthy male adults (international male students)	Daily fasting of 16 h. In the TRF group, participants were allowed to consume their normal diet with no food restriction for only 8 h per 24 h, that is, from 19.30 to 03.30 h for 25 days.	The control group continued their regular diet and were not given any specific instructions or time restriction.	25 days
Gabel et al. Effect of time restricted feeding on the gut microbiome in adults with obesity: a pilot study, 2020.	USA	Pilot study (one group only)	23 (3/20)*	Inclusion criteria: 25–65 years 50 ± 2 (mean ± SEM)*	Adults with obesity Inclusion criteria: BMI 30–45 kg/m^2^	During the TRF intervention, participants ate *ad libitum* from 10:00 to 18:00 daily, and fasted from 18:00 to 10:00 daily. During the feeding window (8 h), there were no restrictions on types or quantities of foods consumed and participants were not required to monitor calorie intake. During the fasting period (16 h), only water and calorie-free beverages were allowed.	None	2-week baseline period followed by a 12-week TRF intervention period.
Ozkul et al., Structural changes in gut microbiome after Ramadan fasting: a pilot study, 2020.	Turkey	Pilot study (one group only)	9 (2/7)	Inclusion criteria: >18 years of age Age range was between 31-56 years (45.0 ± 9.7)	Healthy adult volunteers Inclusion criteria: normal BMI	The study was conducted during the 2015 Ramadan (between 18 June and 16 July), consisting of approximately 17 h of fasting per day during a 29-day period.	None	29 days
Ali et al., Ramadan fasting leads to shifts in human gut microbiota structured by dietary composition, 2021.	China	Non-randomized trial	34 (16 Chinese and 18 Pakistani) Sex (M/F) distribution not stated	Inclusion criteria: 18-40 years Participant age range or mean ± SD age not stated	Two ethnic groups living in close regional proximity: Chinese and Pakistani. All were healthy adult participants.	All participants attended Ramadan fasting from May 15, 2018, to June 15, 2018, in which no food or beverages are consumed between sunrise to sunset for 29–30 days.	None	30 days
Zeb et al., Time-restricted feeding is associated with changes in human gut microbiota related to nutrient intake, 2020.	China	Non-randomized controlled trial	total: 30 (30/0) TRF: 15 (15/0) control: 15 (15/0)	Age range: 18 to 38 years TRF: 27.51 ± 5.84 control: 26.13 ± 2.38	Healthy male students	In the TRF group participants only consumed food in a time-restricted framework (i.e., from 19.30 to 03.30; nocturnal feeding). There was no restriction on food quality, variety, or quantity. Furthermore, the TRF group fasted for 16-h/day for 25 days.	The control group had no time restriction nor any restrictions on food quality, variety, or quantity. They were allowed to consume food throughout the day and night.	25 days
Su et al., Remodeling of the gut microbiome during Ramadan-associated intermittent fasting, 2021.		This article consisted of two separate cohorts, which are addressed separately below.						
Su et al., Young male adult cohort.	China	Uncontrolled trial (one group only)	42 (42/0)	Mean ± SD age for the 30 individuals who completed the intervention was stated as 18.63 ± 1.75. However, the age of those 42 individuals who originally started the intervention was not stated.	Healthy, nonobese young men Inclusion criteria: BMI between 18.5 and 29.9 kg/m^2^	The study was conducted during 2016 Ramadan and consisted of 30 days of Ramadan fasting. Fasting period of each day was from dawn to sunset, which was approximately 16 h in this study.	None	30 days
Su et al., Middle-aged cohort.	China	Non-randomized controlled trial	total: 37 (17/20) TRF: 27 (10/17) control: 10 (7/3)	TRF: 39.9 ± 6.4 control: 42.6 ± 7.9	Healthy, nonobese middle-aged	The study was conducted during 2018 Ramadan and consisted of 30 days of Ramadan fasting followed by 30 days of *ad libitum* diet. During Ramadan the daily fasting period was 16 h.	*Ad libitum* diet for the whole study duration (60 days).	60 days

* This information can be found in Gabel et al. (25). Gabel et al. (26) is a secondary analysis of this study. Abbreviations in alphabetical order: ADF = alternate day fasting, RCT = randomized controlled trial, SD = standard deviation, SEM = standard error of the mean, TRF = time-restricted eating.

### 3.2 Risk of bias in studies

Risk of bias assessments for each included study are presented in Tables 2A,B. RCTs evaluated using Cochrane’s RoB 2 tool (22) are presented in Table 2A, and non-randomized studies evaluated using Cochrane’s ROBINS-I tool (23) are presented in Table 2B. One out of three RCTs (27) evaluated using RoB 2 tool was evaluated having overall high risk of bias due to deviations from the intended interventions. Other two RCT studies (8, 28) were evaluated having some concerns regarding overall risk of bias due to the domain” Selection of the reported result” of the tool. Out of the five non-randomized trials evaluated using ROBINS-I tool, all were evaluated having no information regarding the overall risk of bias due to having no information regarding the domains” Bias due to confounding” and” Bias in selection of the reported result.” No information was selected due to the tool holding questions in these domains that were not applicable to these studies. As to the rest domains of bias, three of the studies (20, 26, 29) were evaluated having a low risk of bias for all, and two studies (30, 31) were evaluated having a moderate risk of bias in the “Bias due to missing data” domain but a low risk of bias in the remaining domains.

**Table 2:** Risk of bias in studies.

(A) Risk of bias assessments for randomized controlled trials evaluated using Cochrane’s ROB2 tool							
Study (First author, title, year of publication)	Risk-of-bias tool	Domain 1: risk of bias arising from the randomization process	Domain 2: risk of bias due to deviations from the intended interventions (effect of assignment to intervention)	Domain 3: missing outcome data	Domain 4: risk of bias in measurement of the outcome	Domain 5: risk of bias in selection of the reported result	Overall risk of bias
Guo et al., Intermittent fasting improves cardiometabolic risk factors and alters gut microbiota in metabolic syndrome patients, 2021.	RoB 2	Low	Low	Low	Low	Some concerns	Some concerns
Cignarella et al., Intermittent fasting confers protection in cns autoimmunity by altering the gut microbiota, 2018.	RoB 2	Some concerns	Low	Low	Low	Some concerns	Some concerns
Zeb et al., Effect of time-restricted feeding on metabolic risk and circadian rhythm associated with gut microbiome in healthy males, 2020.	RoB 2	Some concerns	High	Low	Low	Some concerns	High

(B) Risk of bias assessments for non-randomized trials evaluated using Cochrane’s ROBINS-I tool									
Study (first author, title, year of publication)	Risk-of-bias tool	Bias due to confounding	Bias in selection of participants into the study	Bias in classification of interventions	Bias due to deviations from intended interventions	Bias due to missing data	Bias in measurement of outcomes	Bias in selection of the reported result	Overall bias
Gabel et al., Effect of time restricted feeding on the gut microbiome in adults with obesity: a pilot study, 2020	ROBINS-I	No information	Low	Low	Low	Low	Low	No information	No information
Ozkul et al., Structural changes in gut microbiome after Ramadan fasting: a pilot study, 2020.	ROBINS-I	No information	Low	Low	Low	Low	Low	No information	No information
Ali et al., Ramadan Fasting Leads to Shifts in Human Gut Microbiota Structured by Dietary Composition, 2021.	ROBINS-I	No information	Low	Low	Low	Low	Low	No information	No information
Zeb et al., Time-restricted feeding is associated with changes in human gut microbiota related to nutrient intake, 2020.	ROBINS-I	No information	Low	Low	Low	Moderate	Low	No information	No information
Su et al., Remodeling of the gut microbiome during Ramadan-associated intermittent fasting, 2021.	This article consisted of two separate cohorts, which are addressed separately below.								
Su et al., Young male adult cohort.	ROBINS-I	No information	Low	Low	Low	Low	Low	No information	No information
Su et al. Middle-aged cohort, 2021	ROBINS-I	No information	Low	Low	Low	Moderate	Low	No information	No information

### 3.3 The effects of IF on gut microbiota

Characteristics of the methodology used for gut microbiota composition and diversity assessments—including the time points of fecal sample collection and the used alpha and beta diversity indices—as well as the observed changes in alpha and beta diversity, can be found in Table 3. Overall, six (8, 20, 26, 27, 29, 31) out of eight studies measured the richness and/or alpha diversity, five (8, 20, 27, 29, 31) out of eight studies beta diversity, and all eight studies (8, 20, 26–31) measured microbiota composition. Notably, only two studies (26, 31) reported numerical values for alpha diversity indices, whereas other studies expressed this information in diagrams only.

**Table 3:** Characteristics of the methodology used for gut microbiota composition and diversity assessments.

Study reference	Fecal sample collection time points	DNA extraction kit/method	Gut microbiota amplification region and sequencing platform used	Taxonomical classification	Gut microbiota diversity assessment measures		Change in diversity	
					α-diversity index	β-diversity index	α-diversity index	β-diversity index
Guo et al. (8)	Sample collections were performed at baseline and at 8 weeks the day after a non-fasting day.	Qiagen QIAamp 99 DNA Stool Mini Kit (Qiagen)	16S rRNA regions 16SV4, 16SV3, 16SV3-V4, 16SV4-V5 Illumina NovaSeq	OTU	Shannon, Simpson, Observed species (OTUs)	Unweighted and weighted UniFrac, PCoA	NS for all indices	Unweight UniFrac: significant shift in the intervention group Weighted UniFrac: NS
Cignarella et al. (28)	All patients provided stool samples before initiating steroids (baseline/day 1 visit) and on the day 15 visit.	PowerSoil DNA Isolation Kit (Mo Bio Laboratories, Carlsbad, CA, USA)	16S rRNA V1–V3 region Illumina Miseq v3 platform (2 × 300 bp paired-end reads)	OTU	Alpha diversity was not measured	Beta diversity was not measured	-	-
Zeb et al. (27). Healthy males	From both groups, stool samples were collected only once after 25 days of trial.	Power Soil DNA Isolation Kit (Mo Bio Laboratories)	16S rRNA V1–V3 region Illumina Miseq v3 platform (2 × 300 bp paired-end reads)	OTU	Microbial richness	PCA, Bray–Curtis dissimilarity	Significantly higher in the intervention group after trial	Significantly different between the intervention group and control group after trial
Gabel et al.(26)	Stool samples were collected three times: at B1 (beginning of the baseline period), at W1 (first day of intervention) and at W12 (after 12 weeks of TRF).	Not stated	16S rDNA V4 region Not stated	OTU	Shannon, OTU Richness	Beta diversity was not measured	NS for all indices	-
Ozkul et al.(20)	Stool samples were collected at the start and right after the end of the Ramadan.	QIAamp DNA Stool Mini Kit (Qiagen, Hilden, Germany)	16S rRNA V4 region Illumina MiSeq 2 × 150 bp platform	OTU	Microbial richness (Observed OTUs), Phylogenetic diversity, Shannon index	Unweighted and weighted UniFrac	Observed OTU’s ↑ Phylogenetic diversity NS Shannon index NS	Unweighted UniFrac: significant shift Weighted Unifrac: significance unclear
Ali et al.(29)	Sample collection before fasting was conducted on the morning of May 15, 2018 (first day of Ramadan), while samples after fasting were retrieved on the morning of June 15, 2018 (last day of Ramadan).	QIAamp DNA Stool Mini Kit, QIAGEN, Hilden, Germany	16S rRNA V3 and V4 region MiSeq Reagent Kit v3 (Illumina, San Diego, CA, United States)	OTU	Chao1, Observed species, Observed OTUs, ACE, Coverage index, Shannon, Simpson	PCoA based on Bray-Curtis distances	Chinese: ACE ↑ Coverage index ↓ NS for other indices Pakistani: NS for all indices	Chinese: Slight shift Pakistani: Substantial shift
Zeb et al.(30). Nutrient intake	Stool samples were collected from both groups after 25 days of TRF or non-TRF.	PowerSoil PowerLyzer DNA Isolation Kit in conjunction with the PowerLyzer 24 homogenizer (both MO BIO Laboratories, Inc.; Carlsbad, CA, USA)	16S rRNA V3-V4 region Illumina MiSeq platform (2 × 250 bp, paired-end sequenced)	OTU	Alpha diversity was not measured	Beta diversity was not measured	-	-
Su et al. (31).	This article consisted of two separate cohorts, which are addressed separately below.							
Su et al. (31). Young male adult cohort	Stool samples were collected three times: at T1 (day 0; the first day of the 2016 Ramadan), at T2 (day 15) and at T3 (day 30; the final day of Ramadan).	QIAamp DNA Stool Mini Kit (Qiagen)	Next-generation sequencing of the V3–4 region of the bacterial 16S rRNA gene was performed by the NovoGene Company	OTU	Shannon, Simpson, Chao1, ACE, PF whole tree, Goods coverage	PCoA based on the Bray-Curtis distance	Shannon ↑ Simpson ↑ Chao ↑ ACE ↑ PF whole tree↓ Goods coverage NS	Significant shift
Su et al. (31). Middle-aged cohort	Stool samples were collected three times: at T1 (day 0; start of the 2018 Ramadan), at T2 (day 30; end of the 2018 Ramadan) and at T3 (day 60; 1 month after the end of Ramadan fasting).	QIAamp DNA Stool Mini Kit (Qiagen)	Next-generation sequencing of the V3–4 region of the bacterial 16S rRNA gene was performed by the NovoGene Company	OTU	Shannon, Simpson, Chao1, ACE, PF whole tree, Goods coverage	PCoA on Bray-Curtis dissimilarities of bacterial communities	NS for all indices	Significant shift in the intervention group NS in control group

ACE = abundance-based coverage estimator, NS = not statistically significant, OTU = operational taxonomic unit, PCA = principal component analysis, PCoA = principal coordinate analysis, rRNA = ribosomal RNA, UniFrac = unique fraction metric.

#### 3.3.1 Richness and alpha diversity

In healthy males, microbial richness was significantly higher in the TRF group than in the control group in the end of the 25-day intervention period (linear regression *p* < 0.005) (27). In obese adults, operational taxonomic units (OTU) Richness, which is the sum of unique OTUs found in each sample, was not significantly different between beginning of the baseline period (B1) (436 ± 105), first day of the intervention (W1) (459 ± 115), and at 12 weeks of TRF (W12) (460 ± 119) (26). Shannon Diversity was not significantly different between B1 (3.81 ± 0.40), W1 (3.88 ± 0.41), and W12 (3.97 ± 0.41), either (26).

Among healthy Chinese participants, comparisons between pre- and post-Ramadan showed that the abundance-based coverage estimator (ACE) was higher in the end of Ramadan (*p* = 0.026), whereas the coverage index was lower in the end of Ramadan (*p* = 0.039) (29). Other alpha diversity indices showed no significant differences between pre- and post-Ramadan within neither Chinese nor Pakistani group (29). In another study conducted on healthy adults, unpaired t-test revealed a significant increase in microbial richness (Observed OTUs) post-Ramadan compared to baseline levels (*p* = 0.016), while no significant difference was observed between the two time points in terms of phylogenetic diversity (*p* = 0.052) and Shannon index (*p* = 0.121) (20).

In a healthy young cohort without a control group, there was a significant increase in Shannon (5.17 ± 0.49 vs. 5.40 ± 0.44; *p* = 0.02), Simpson (0.92 ± 0.04 vs. 0.94 ± 0.02; *p* = 0.001), Chao1 (350.9 ± 34.5 vs. 372.4 ± 55.5; *p* = 0.04) and ACE (354.4 ± 34.9 vs. 378.6 ± 56.4; *p* = 0.01) alpha diversity indices following Ramadan, whereas PF whole tree decreased (48.68 ± 11.40 vs. 37.64 ± 10.20; *p* < 0.001) (31). As for the Good’s Coverage alpha diversity index, there was no significant difference between pre- and post-Ramadan (0.99 ± 0.0002 vs. 0.99 ± 0.0002; *p* = 1) (31). In a separate healthy middle-aged cohort reported in the same paper, the Shannon index of the TRF group showed a slight upward trend, both compared with controls (values not reported) as well as compared with the pre-TRF state (5.04 ± 0.59 vs. 5.13 ± 0.59; *p* = 0.46), but this effect was not statistically significant. Likewise, no significant differences were observed in the other alpha diversity indices in the middle-aged TRF group after 30 days of Ramadan; Simpson (0.92 ± 0.06 vs. 0.92 ± 0.06; *p* = 0.70), Chao1 (352.0 ± 34. vs. 3342.9 ± 39.7; *p* = 0.31), ACE (350.7 ± 31.1 vs. 342.8 ± 35.4; *p* = 0.34), PF whole tree (21.99 ± 2.10 vs. 21. 69 ± 2.18; *p* = 0.62) and Good’s Coverage (0.99 ± 0.0005 vs. 0.99 ± 0.0004; *p* = 0.12) (31).

In the sole study that covered ADF, the authors did not measure the alpha diversity (28). The only 5:2 diet study, which was conducted on metabolic syndrome patients, showed no significant differences in the alpha diversity at baseline between the 5:2 diet group and control group (8). The number of observed species (OTUs) did not differ significantly between baseline and post-intervention within each group. No statistically significant changes in alpha diversity in the 5:2 diet group were observed with the Shannon index (*p* = 0.983) or Simpson index of 1-D (*p* = 0.977).

#### 3.3.2 Beta diversity

The study with healthy males (27) applied a principal component analysis (PCA) to illustrate microbiome similarity in the TRF and control groups at the OTU level. Samples from the two groups clustered separately, indicating that the groups had two distinct microbiome communities in the end of the 25-day intervention period (permutational multivariate analysis of variance (PERMANOVA) test, *p* < 0.05).

In healthy adults, microbial community structure (β-diversity) was significantly different between pre- and post-Ramadan [unweighted unique fraction metric (UniFrac) analysis *p* = 0.025] (20). Likewise, principal coordinate analysis (PCoA) using the Bray-Curtis model showed that the microbial community composition exhibited substantial divergence with little overlap between pre- and post-Ramadan in healthy Pakistani participants (29). However, in healthy Chinese participants, microbial community composition shifted only slightly following Ramadan (29). Subsequent PERMANOVA tests further supported the significant differences in the Pakistani group in the beginning vs. in the end of Ramadan (*p* = 0.0129) (29).

In the heathy young cohort, the structure of gut microbiota differed significantly between day 0 (the beginning of Ramadan) and day 30 (the end of Ramadan) (analysis of similarities (ANOSIM) test, *p* < 0.001) (31). Similarly, in the healthy middle-aged cohort TRF group, gut microbiota structures differed significantly between T1 (the beginning of Ramadan; day 0) and T2 (the end of Ramadan; day 30) (ANOSIM test, *p* < 0.001) (31). Unlike the young cohort, the middle-aged cohort additionally included a 30-day *ad libitum* follow-up period after the cessation of fasting. Interestingly, the gut microbial community showed a significant trend (ANOSIM test, *p* < 0.001) of return toward baseline conditions after the discontinuation of fasting (T3; 1 month after the end of Ramadan), indicating that the effects of TRF are reversible (31). As for the non-fasting control group of the middle-aged cohort, no significant differences were found between T1, T2 and T3, i.e., microbiome composition did not change during the study period (31). The authors pointed out that this observation agreed with the notion that gut microbiomes tend to be stable when lifestyles are not changed.

In the sole study that covered ADF, the authors did not measure the beta diversity (28). In the 5:2 diet study with metabolic syndrome patients (8), PCoA of unweighted UniFrac distances based on OTU data from the phylotype sequencing run showed a significant shift in the microbial community compositions in the end of the intervention within the 5:2 diet group (5:2 diet baseline vs. 5:2 diet 8 weeks, *p* = 0.005). Conversely, PCoA of weighted UniFrac distances based on OTUs data and the relative abundances showed no significant alteration in the composition of the gut microbiota within either the 5:2 diet group or the control group.

#### 3.3.3 Gut microbiota composition

Statistically significant changes in the gut microbiota composition are presented in Supplementary Table S1A (the beginning vs. the end of trial comparisons of fasting groups), in Supplementary Table S1B (the beginning vs. the end of trial comparisons of control groups), and in Supplementary Table S1C (the end of trial comparisons of fasting vs. control groups). Guo et al. (8), Gabel et al. (26), Ozkul et al. (20), and Ali et al. (29) as well as both the young and the middle-aged cohort of Su et al. (31) compared the gut microbiota compositions of fasting groups in the beginning vs. in the end of the intervention period. Studies that compared the gut microbiota compositions of control groups in the beginning vs. in the end of intervention period included Guo et al. (8) and the middle-aged cohort of Su et al. (31). Both studies by Zeb et al. (27, 30) compared the gut microbiota compositions of fasting groups to those of control groups in the end of the intervention period. Lastly, Cignarella et al. (28) made all three kinds of comparisons.

There was a lot of heterogeneity in the compositional changes observed in fasting groups (Supplementary Table S1A) and the bacteria which were significantly affected by IF were mostly different in each study. Additionally, if a significant change in certain bacterial taxa’s abundance was observed in multiple studies, the changes were sometimes opposite in direction. At phylum level, for example, Bacteroidetes were found increased in Ozkul et al. (20) and the Pakistani group of Ali et al. (29), but conversely, decreased in the Chinese group of Ali et al. (29) and the young cohort of Su et al. (31). Similarly, contradicting results were found regarding Firmicutes, as they were found increased in the young cohort of Su et al. (31), but decreased in Ozkul et al. (20) and the Pakistani group of Ali et al. (29). Likewise, there was some discrepancy concerning Proteobacteria, as they were increased in the Chinese group as well as the total participants (Chinese+Pakistani) of Ali et al. (29) and in the young cohort of Su et al. (31) but decreased in Ozkul et al. (20). The study by Gabel et al. (26) is not mentioned in Supplementary Table S1A, as it showed no significant differences in community composition between B1, W1, and W12.

By considering only bacteria whose abundance significantly increased/decreased in three or more fasting groups, the abundance of Proteobacteria (at phylum level), Gammaproteobacteria (at class level), Clostridiales (at order level) and *Faecalibacterium* (at genus level) were increased. Meanwhile, Negativicutes (at class level), Selenomonadales (at order level) and *Veillonellaceae* (at family level) were decreased.

Regarding potential compositional changes in control groups (Supplementary Table S1B), in Guo et al. (8) a linear discriminant analysis (LDA) showed that some bacterial taxa were differentially abundant between baseline and 8 weeks within the control group (LDA-score > 3.00). Contrastingly, in the middle-aged cohort of Su et al. (31), LDA coupled with effect size measurements (LEfSe) showed that there were no differentially abundant taxa in the non-fasting control group either immediately after or one month after the cessation of fasting (LDA-score threshold value 4.00), i.e., no taxa were significantly changed during the study period.

As shown in Supplementary Table S1C, both studies by Zeb et al. (27, 30) found that the fasting and control groups had significant differences in their gut microbiota compositions in the end of the study period. However, since in both these studies the stool samples were collected only once in the end of the trial, it remains unclear whether the groups might have had significant differences in their gut microbiota compositions to begin with.

Cignarella et al. (28) compared the relative abundances of four major phyla, Bacteroidetes, Firmicutes, Actinobacteria and Verrucomicrobia, between the beginning and the end of the intervention period. No bacteria were significantly different at day 15 between the ADF and control group, but the abundance of *Faecalibacterium*, *Lachnospiracea incertae sedis* and *Blautia* showed an increasing trend after 15 days of ADF.

### 3.4 Weight loss and dietary changes in different types of IF studies

At this point, it should be noted that the different forms of IF substantially differ in nature. ADF and 5:2 diet have an energy restriction on the fasting days, meaning that the total energy (kcal/day) and macronutrient intakes (g/day) are reduced on these days. What requires examination, however, is whether besides changing the food quantity, ADF and 5:2 diet could also change the composition/quality of the subjects’ diet, i.e., if the relative proportions of macronutrients (energy%) or consumed foods/food groups change during the ADF and 5:2 diet period compared to baseline. Moreover, the effects of ADF and 5:2 diet on weight/BMI should be considered. A summary of the changes in weight and/or BMI, as well as the changes in energy, macronutrient and food/food group intakes that were observed in the different types of IF studies included in this review is presented in Supplementary Table S2.

In the sole 5:2 diet study of this review (8), weight and BMI decreased significantly in the 5:2 diet group. However, it is difficult to postulate whether the 5:2 diet group modified their diet composition during the intervention period or not based on the limited diet-related information that was reported in the study. Similarly, the sole ADF study (28) observed a decreased BMI in the ADF group but reported only little about participants’ diet.

The nature of Ramadan fasting and other non-religious TRF intervention designs included in this review was such that there were no restrictions on energy intake nor restrictions on food quality or quantity. Another difference with 5:2 diet and ADF, which do not intervene with meal timing, is how TRF interventions change the feeding-fasting pattern by restricting daily food consumption to a specific time window. Ramadan fasting also changes circadian rhythms as participants are only allowed to eat nocturnally, between sunset and sunrise. In other non-religious TRF interventions the feeding-window can occur either at nighttime or daytime.

Out of the six TRF studies included in this review, only Gabel et al. (26) and both the young and the middle-aged cohort of Su et al. (31) seemed to investigate changes in body weight. In all three, TRF led to significant weight loss. Regarding energy intake, only Gabel et al. (26) and Ali et al. (29) and the middle-aged cohort of Su et al. (31) reported information on energy intake both in the beginning and the end of intervention period. Out of the three, Gabel et al. (26) and Su et al. (31) reported significantly decreased energy intake during the intervention, whereas, contrastingly, Ali et al. (29) found no significant differences in energy intake in the beginning vs. in the end of Ramadan among neither Chinese nor Pakistani group.

Regarding information on macronutrient intakes, only Ali et al. (29) and Gabel et al. (25), the secondary analysis of which Gabel et al. (26) was, reported data on macronutrient intakes both in the beginning and in the end of intervention period to allow for a comparison. Additionally, Ali et al. (29) reported information on intakes of certain foods or food groups. In Gabel et al. (25), macronutrient intakes (energy%) remained very similar between B1 and W12. Similarly, Ali et al. (29) did not observe significant changes in macronutrient intakes (energy%) in the beginning vs. in the end of Ramadan within Chinese nor Pakistani group. Despite this, Ali et al. (29) did interestingly observe that within both Chinese and Pakistani group, the intakes of certain food groups were significantly different in the beginning vs. in the end of Ramadan. These decreases and increases in the consumption of certain foods indicate that dietary habits may change during fasting. This should be further investigated in future studies.

## 4 Discussion

### 4.1 General limitations of the evidence included in the review

Comparing the studies included in this review with each other is challenging because of the considerable heterogeneity in study populations, study and intervention designs, and indices used to measure the same outcomes. All the aforementioned factors make it challenging to draw any general conclusions on how exactly IF affects the gut microbiota. In order to achieve this, more studies conducting a certain type of intervention in a certain population and measuring the same outcomes in the same way are definitely needed.

Furthermore, a wide generalization of the results obtained from one specific population or directly applying the results from one population to another may not be reasonable. It is known that along with diet (32, 33), genes (34), environment (35), ethnicity (36) and age (37) all shape the gut microbiota, so the IF-induced changes may not be the same in different populations. A difference in TRF’s impact on gut microbiota was seen in Ali et al. (29) which included two different ethnic groups living in close regional proximity. The study featured a 24-h dietary recall for 3 days and a food frequency questionnaire prior and post intervention. It was seen that the proportions of energy sources (% of total energy intake) consumed by each group were significantly different between the Chinese and Pakistani groups. The Chinese participants consumed significantly more carbohydrates, while the Pakistani group consumed significantly more fats and proteins.

Substantial limitations of the evidence included in the review concern sample size and study designs. The sample sizes of the studies ranged from *n* = 9 to *n* = 80, which can be considered relatively small. As RCTs are generally recognized as the gold-standard for studying causal relationships between an intervention and outcome (38), one notable limitation is that only three out of the eight included studies were RCTs, while the rest five represented different types of quasi-experimental study designs. The weakness of quasi-experimental studies may be confounding, which is addressed in detail in the next section.

Moreover, another limitation related to study designs is that many of the studies were, besides non-randomized, also uncontrolled. The lack of a control group makes it difficult to definitely prove that observed pre-post changes were caused by the IF intervention in particular and would not have happened during the study period otherwise. However, in case of gut microbiota studies it can be argued that a lack of control group may be justified. As stated in Ozkul et al. (20), although the absence of non-fasting control group prevents the assessment of possible minor changes in the gut microbiota during the study period, the gut microbiota of adult subjects is relatively stable over time. This notion is somewhat supported by the results obtained in Guo et al. (8) and the middle-aged cohort of Su et al. (31), which showed some and no compositional changes, respectively, in the control group during the study period. Nonetheless, RCTs are needed to gain more reliable evidence of IF’s impact on the gut microbiota.

### 4.2 Confounding

#### 4.2.1 Identification of relevant confounding domains

Potential confounders were identified based on both existing knowledge of the literature and discussions between the members of our review group. Using the largest publicly available 16S dataset of the gut microbiota [from the American Gut Project (AGP)], Vujkovic-Cvijin and colleagues have listed the following potential confounders which have a strong association with the composition of the gut microbiota: BMI, sex, age, geographical location, frequency of alcohol consumption, bowel movement quality, and dietary intake frequency of various food types (39). In addition to these, ethnicity, use of pre- or probiotics, use of antibiotics and socioeconomic status were identified as potential confounders.

Because some confounding domains may not be directly measured, investigators can measure specific variables in an attempt to fully or partly adjust for these confounding domains (40). In case of socioeconomic status, income and education can be used to adjust for it, as it cannot be directly measured. As diet is a central factor that affects gut microbiota composition (32, 33), socioeconomic status was included on the list of confounding domains with the assumption that it influences dietary choices.

In this review ‘diet’ was defined as a confounding domain by the following measurable variables: energy intake, macronutrient intake (fat, carbohydrate, protein, dietary fiber), and the intake of different foods/food groups. It should be noted that these variables are often measured with questionnaires, interviews or food diaries, i.e., with methods that are based on self-report and, if retrospective, rely on memory. This might lower the reliability of these measures.

Regarding confounding due to diet, there are a few things to note. To avoid baseline confounding, it would be best if the IF and control group had similar diets, so that the groups would be as comparable as possible to begin with. Furthermore, during the intervention, it would be important that the groups maintain a similar diet compared to baseline and also compared to each other. This would facilitate distinguishing the independent effect of IF itself on the gut microbiota, which may be confounded by between-group differences or within-group changes in diet composition/quality.

#### 4.2.2 Bias due to confounding

One central difficulty in attempting to draw conclusions about the causal relationship between IF and gut microbiota richness, alpha and beta diversity, and composition based on the studies included in this review, is the bias due to confounding. As stated earlier, majority of the studies in this review were quasi-experimental. Despite this, the authors of the original studies rarely addressed concerns related to confounding, i.e., what confounders they controlled for, if any, or what method they used for this, hence making it difficult from our side to judge whether an appropriate analysis method that controlled for all the important confounders was used. Thus, it is very difficult to evaluate to what extent bias due to confounding may be present in the studies, and whether the observed intervention effects truly arose from the intervention itself or if they were at least partly explained by confounding factors.

### 4.3 Possible mechanisms behind the IF-induced changes in gut microbiota

#### 4.3.1 Weight loss and dietary changes

In many—although not all—studies included in this review, IF induced changes in gut microbiota richness, alpha and beta diversity, and composition. It is thus important to aspire to understand the underlaying mechanism(s) explaining these changes. We propose that the changes could be, at least in part, driven by weight loss or dietary changes that may happen during an IF intervention, as both weight and diet substantially affect gut microbiota.

Body weight and adiposity have been reported to affect the Firmicutes-Bacteroides ratio with individuals with obesity having a greater Firmicutes/Bacteroidetes ratio (41, 42). It has also been found that individuals with obesity have other compositional differences compared with lean individuals (41). Diet and dietary components can strongly influence the gut microbiota composition and are among the most important contributors to its alteration (32, 33).

Based on the studies included in this review, it is difficult to conclude to which extent IF-induced changes in the gut microbiota may be explained by changes in body weight and/or BMI. In future IF studies analyses need to be performed to examine whether weight loss has any significant mediating effect on the changes in gut microbiota richness, alpha and beta diversity, and composition.

As for dietary information, apart from Gabel et al. (25) and Ali et al. (29), the studies did not report pre- and post-intervention dietary measurements in sufficient detail to be able to evaluate whether diet composition/quality changed during IF, and if this could at least partly explain the observed changes in gut microbiota. Furthermore, as the observations by Ali et al. (29) point out, substantial changes in the foods/food groups consumed are not necessarily reflected on the macronutrient intakes, as even very similar macronutrient intakes can consist of very different foods. Thus, it would be advisable that future IF studies collect and report not only energy and macronutrient intakes of the participants, but also the intakes of specific foods and/or food groups.

Further research is also required about possible gender differences regarding IF interventions and possible resulting weight loss and gut microbiota changes. In a study by Domaszewski et al. (43) it was seen that TRF may have gender-related effects on body composition and that gender differences could be driven by sex specific differences in insulin and adrenaline secretion after fasting. A recent study by Khan et al. (7) evaluated the impact of TRF on BMI and gut microbiota outcomes in groups of overweight/obese, normal weight and underweight males and females. Interestingly, female subjects who were underweight gained weight during TRF intervention, showing for the first time that IF regimen could potentially normalize body weight. More research about the relation of gender and gut microbiota changes in response to IF is needed.

#### 4.3.2 Changes in feeding-fasting pattern and circadian rhythms

As stated in the previous sections, different forms of IF seem to induce changes in gut microbiota richness, alpha and beta diversity, and composition, and might also cause changes in body weight/BMI and energy, macronutrient and food/food group intakes. However, based on the evidence included in this review, it cannot be said to which extent the latter may explain the former.

One question that arises is whether IF would still affect gut microbiota-related outcomes if the subjects’ weight and diet composition remained the same, and which could be the mechanism behind this. At least for TRF studies it could be possible that the change in feeding-fasting pattern would be enough to independently provoke changes in the gut microbiota. A mice study has demonstrated that TRF affected the gut microbiota composition even when mice were fed with the same diet (44). In this study, mice received the same feed with either *ad libitum* access or during an 8-h feeding window. Future studies are needed to confirm this observation in humans.

In Western culture, the normal daily meal distribution is three to five meals, spread from breakfast to late dinner (45), which means that energy and nutrients are constantly available for the gut microbiota. Furthermore, it has been reported that an overnight fast of 8-10 h is normal for most people (46). In TRF, the fasting interval – whether it occurs at day or night – is considerably longer, e.g., 16 h. Hypothetically, this could favor the growth of certain bacteria. Ozkul et al. (20) found that the species *Akkermansia muciniphila* was enriched in the end of Ramadan fasting. It was stated that as a mucin degrading bacterium, *A. muciniphila* strongly adheres to the mucus layer, and can resist environmental changes, such as decreased food intake and changes in the intestinal flow rate. In the middle-aged cohort of Su et al. (31), the family *Lachnospiraceae* were increased in the end of Ramadan fasting. The authors reported that many members of this family appear to have the capacity to ferment mucins and suggested that this ability provides *Lachnospiraceae* a competitive advantage during IF when other carbohydrates are unavailable as an energy source to the gut microbiota for extended time periods.

Concerning circadian rhythms, Ozkul et al. (20) point out that the feeding-fasting pattern in Ramadan fasting is not compatible with human circadian rhythms. Future human studies are needed to investigate the role of either maintaining or disrupting daily circadian rhythms in TRF interventions, and whether changes in the gut microbiota may result from changes in circadian rhythms.

### 4.4 Limitations of the review process

Originally, when the review was registered to PROSPERO (RRID:SCR_019061), the plan was to conduct a meta-analysis on this topic. However, the heterogeneity of the studies and the use of different indices to measure the same outcomes (e.g., the variety of different indices used to measure alpha diversity), as well as the lack of reporting numerical values for diversity indices and composition-related measures [such as OTU number of bacterial taxa or relative abundance (%) of bacterial taxa] prevented us from doing so.

Overall, the limited number of papers available during the literature search can be seen as a limitation for the review process. The aim of this systematic review was to gain understating of how different types of IF interventions (TRF, ADF and 5:2 diet) affect gut microbiota richness, alpha and beta diversity, and composition. However, only one study being available for ADF and 5:2 diet each made it impossible to reasonably compare the different IF subtypes and draw any valuable conclusions on how their impacts on gut microbiota may differ.

The literature search was conducted in the beginning of the review process and includes studies published at that time. Therefore, this systematic review does not include the latest publications on the topic. According to a quick, non-systematic literature search in PubMed, at least two studies have been published since. One is a study by Chen et al. (47), which is continuation to the study by Ali et al. (29) and focuses on analyzing fecal metabolites. The second new study is the TRF study by Khan et al. (7), which was briefly referenced in the introduction and the discussion.

### 4.5 Possible health benefits of IF-induced gut microbiota changes

In this section we discuss whether the effects of IF on gut microbiota diversity and composition might be beneficial for human health, with a particular focus on their significance to inflammatory bowel disease (IBD) and irritable bowel syndrome (IBS).

#### 4.5.1 Alpha diversity

Alpha diversity, the microbial diversity of an ecological community, is the most common indicator for assessing gut microbiota health in adults (48). In general, a high diversity provides the ecosystem with strong stability as well as its ecological function (49). Higher alpha diversity has also been associated with numerous health indicators suggesting that gut microbiota heterogeneity may play a role in responses to dietary and lifestyle interventions (50). Meanwhile there is mounting evidence of lower diversity levels being observed in several acute and chronic illnesses (51). A higher diversity microbiota typically includes large proportions of anaerobes, whereas when diversity is reduced, facultative anaerobes, including phyla such as Proteobacteria and Bacilli, increase (52).

#### 4.5.2 Compositional changes

For compositional changes, the bacteria with most consistent evidence are focused herein, i.e., the bacteria whose abundance significantly increased/decreased in three or more fasting groups. As previously mentioned in the results section, the abundance of Proteobacteria (at phylum level), Gammaproteobacteria (at class level), Clostridiales (at order level) and *Faecalibacterium* (at genus level) were increased. Meanwhile, Negativicutes (at class level), Selenomonadales (at order level) and *Veillonellaceae* (at family level) were decreased.

##### 4.5.2.1 Negativicutes, Selenomonadales, and *Veillonellaceae*

The class Negativicutes contains the orders Selenomonadales and Veillonellales, and within the latter belongs the family *Veillonellaceae*. It has been observed that adult patients with IBS had a higher proportion of *Veillonellaceae* in stool than healthy controls (53). Similarly, another study found that *Veillonellacea*e and Negativicutes were significantly enriched in colonic mucosal microbiota samples from adult IBS patients compared to healthy controls (54).

As for IBD, increased amounts of *Veillonellaceae* and Negativicutes were observed in fecal samples of both Crohn’s disease (CD) and ulcerative colitis (UC) patients relative to healthy controls (55). A systematic review comparing the gut microbiota of pediatric patients with IBD to patients without IBD has been published recently (56). This review included 30 studies that investigated gut microbiota profiles from pediatric patients with CD, majority of which reported microbial data from fecal samples. Out of these 30 studies, *Veillonellaceae* were increased in 4. Furthermore, the review included 15 studies which evaluated the gut microbiota profile in pediatric patients with UC, of which 9 assessed feces-associated microbiota, 5 mucosa-associated microbiota, and 1 duodenal fluid-associated microbiota. Out of these 15 studies, *Veillonellaceae* were increased in 1 and decreased in 1.

Due to its increased abundance in the aforementioned diseases, *Veillonellaceae* can be considered to be a pro-inflammatory family of bacteria (57). Therefore, the significant IF-induced decreases in *Veillonellaceae* and Negativicutes may be considered as beneficial changes.

##### 4.5.2.2 Proteobacteria and Gammaproteobacteria

An increased prevalence of the bacterial phylum Proteobacteria has been proposed as a marker of dysbiosis and a potential diagnostic criterion for disease (58). Several studies have observed an increased abundance of members belonging to Proteobacteria in metabolic disorders and IBD (59). Gammaproteobacteria is a class within the phylum Proteobacteria. Gammaproteobacteria are considered proinflammatory due to the production of lipopolysaccharide (LPS), an inflammatory endotoxin (60, 61).

A study conducted on pediatric patients found that children with IBS had greater proportions of the phylum Proteobacteria in their stool than healthy children, which was mainly explained by significantly greater percentage of the class Gammaproteobacteria in IBS patients (62). A systematic review including 16 studies found that a higher proportion of Proteobacteria in the fecal microbiota was associated with IBS in several studies (63). Another systematic review (64) was conducted on studies comparing the fecal or colon microbiomes of adult or pediatric patients with IBS with those of healthy individuals. Out of the total 24 included studies, 4 studies reported increased amounts of Proteobacteria in IBS patients, while 2 studies showed no difference.

In adult patients with IBD, Proteobacteria and Gammaproteobacteria were significantly increased in the fecal samples from ICD (Crohn disease localized in the ileum) patients relative to healthy patients (65). In the earlier mentioned systematic review (56), which included 30 studies conducted on pediatric patients with CD and 15 studies on pediatric patients with UC, Proteobacteria were increased in 3 and decreased in 1 CD studies, Gammaproteobacteria were increased in 3 CD studies, Proteobacteria were increased in 2 and decreased in 1 UC studies, and Gammaproteobacteria were increased in 1 and decreased in 1 UC study.

Another systematic review contained 143 studies that compared adult IBD patients’ gut microbiota from fecal, intestinal lavage or intestinal tissue samples to that of non-IBD controls (66). The increase in the phylum Proteobacteria was extensively reported in IBD patients, for both CD and UC. The review also reported increased Gammaproteobacteria for 3 CD studies.

Considering the above-mentioned evidence, IF-induced increase in Proteobacteria and Gammaproteobacteria may be considered harmful.

##### 4.5.2.3 Clostridiales and *Faecalibacterium*

*Faecalibacterium* is one of the major butyrate producers in the intestine (67). Butyrate has been reported to exert anti-inflammatory effects, such as inhibiting numerous proinflammatory cytokines. Inhibition of NF-κB activation and upregulation of PPAR-γ have been proposed as the mechanisms underlying butyrate’s anti-inflammatory properties. Furthermore, butyrate enhances intestinal barrier function and mucosal immunity (68).

A study comparing the gut microbiota of adult patients with relapsing–remitting multiple sclerosis (RRMS) with that of healthy controls found that species belonging to *Faecalibacterium* were less abundant in the fecal samples of MS subjects than in those of healthy controls (69). Cignarella et al. (28) observed a trend towards increased abundance of *Faecalibacterium* in relapsing MS patients after 15 days of ADF, and suggested that ADF might counterbalance this reported dysbiosis in MS.

The genus *Faecalibacterium*, belonging to the family Ruminococcaceae within the order Clostridiales, contains only one validated species, *Faecalibacterium prausnitzii* (70). *F. prausnitzii* has been found reduced in the gut microbiota of obese individuals (71). Moreover, the abundance of *F. prausnitzii* was significantly decreased in fecal samples from adult CD patients compared with samples from healthy subjects (72). This study is in line with a later meta-analysis concluding that the abundance of *F. prausnitzii* was decreased in IBD patients compared with healthy controls (73).

In the systematic review including 30 studies conducted on pediatric patients with CD and 15 studies on pediatric patients with UC (56), Clostridiales were decreased in 7 CD studies, *Faecalibacterium* were decreased in 8 and increased in 1 CD studies, Clostridiales were decreased in 3 UC studies, and *Faecalibacterium* were decreased in 2 and increased in 1 UC studies. In the systematic review that included 143 studies conducted on adult IBD patients (66), numerous studies reported reduced amounts of *Faecalibacterium* in CD. *F. prausnitzii* was frequently decreased in CD, whereas in UC the results were conflicting.

The systematic review (63), which included 16 studies, found that compared to healthy controls, the relatively consistent changes in the fecal microbiota of IBS patients included an increased abundance of Clostridiales. In another systematic review (64), the genus *Faecalibacterium* (order Clostridiales) was assessed in 4 studies (out of the 24 studies included), decreasing significantly in 3 of them and non-significantly in 1. *F. prausnitzii* was evaluated in 5 studies, where 2 showed a significant decrease, 2 reported an insignificant decrease and 1 showed no difference.

*F. prausnitzii* has been shown to exert anti-inflammatory effects both *in vitro* and *in vivo* (74). Various possible mechanisms have been proposed for *F. prausnitzii* anti-inflammatory effects, not all of them butyrate-related (75). Due to its anti-inflammatory properties, the increase in *Faecalibacterium* during IF may be beneficial for health. According to recent systematic reviews, it seems that *Faecalibacterium* and *F. prausnitzii* are commonly decreased in IBD. Therefore, IF may serve to help counterbalance this dysbiosis.

### 4.6 Implications of the results for practice, policy, and future research

IF is a popular way of eating around the world, and yet little research of its gut microbiota-related benefits for humans exists. Considering our systematic review, its potential effects for gut microbiota modification and weight loss purposes should be further investigated. Once we gain a better understanding of how different forms of IF affect the gut microbiota and its metabolites, IF could be used for enrichment of specific taxa beneficial to gut health and IF interventions could be used to treat and prevent different diseases. Further research on different forms of IF and their potential to serve a therapeutical purpose along with medication for different diseases such as IBD, IBS and metabolic syndrome should be explored.

We would suggest that further research would take dietary behavior both before and during the intervention period into account to exclude diet as a large confounding factor. Well-designed RCTs with larger sample sizes are needed in the future. Lastly, below are listed a few additional aspects related to the relationship between IF interventions and the gut microbiota that require further investigation.

#### 4.6.1 Microbial metabolites

Of the eight studies included in this review, only Guo et al. (8) measured gut microbial metabolites. In this study three circulating gut-derived metabolites were analyzed: lipopolysaccharide (LPS), short-chain fatty acids (SCFAs), and trimethylamine N-oxide (TMAO). It was shown that the 5:2 diet improved plasma SCFAs and plasma LPS but not plasma TMAO.

In future research, it would be important to place more focus on measuring various gut microbiota-derived metabolites, as they are known to have several different local and systemic effects (76, 77). SCFAs, for example, modulate different processes including cell proliferation and differentiation, hormones secretion and activation of immune/inflammatory responses (78). Butyrate, one of the three major SCFAs produced by gut bacteria along with acetate and propionate (79), has been reported to improve gut barrier function by facilitating the assembly of tight junctions (80).

#### 4.6.2 IF duration and frequency, circadian rhythms

In the studies included in this review, the intervention durations ranged from 15 days to 12 weeks. It would be interesting to examine how longer-term IF interventions affect the gut microbiota, or in general, if the effects on gut microbiota differ depending on the IF duration. Furthermore, the resiliency of IF-induced gut microbiota modifications should be studied more. Out of the eight studies in this review, only the middle-aged cohort of Su et al. (31) examined this by including a 30-day *ad libitum* follow-up period after the cessation of fasting. The results of this study suggested that the gut microbiome composition returns to baseline upon cessation of TRF. Thus, it should be further investigated if one has to adopt IF as a continuous lifestyle in order to gain beneficial health effects from it, or if not, how often one should fast (e.g., for one month a few times a year). The role of maintaining or disrupting daily circadian rhythms and the significance of the feeding-window’s timing (in daytime vs. in night-time) also require further investigation, as previously discussed.

## 5 Conclusion

The findings of the present systematic review do not definitely prove that a causal relationship between IF and the improvement of gut microbiota-related outcomes exists, as there were several possible confounding factors as well as limitations to the study designs. It does seem possible, however, that IF can improve richness and alpha diversity and modify the composition of gut microbiota.

In our systematic review, we found that IF may increase the abundances of Proteobacteria, Gammaproteobacteria, Clostridiales and *Faecalibacterium*, and decrease the abundances of Negativicutes, Selenomonadales and *Veillonellaceae*. The potential health benefits of these compositional changes were discussed, with a particular focus on IF’s potential to treat IBS and IBD.

Although some discrepancy exists between different studies, the existing evidence generally suggests an increase in *Veillonellaceae,* Negativicutes, Proteobacteria and Gammaproteobacteria in patients with IBS and IBD, as well as a decrease in *Faecalibacterium* in IBD and potentially also in IBS. Although the IF-induced decrease in *Veillonellaceae* and Negativicutes, as well as the increase in anti-inflammatory *Faecalibacterium,* may therefore be considered as beneficial changes, the increase in proinflammatory Proteobacteria and Gammaproteobacteria, on the other hand, may be harmful. More research is needed to better understand how different forms of IF modify the gut microbiota and further evaluate their possible benefits to human health.

## Data availability statement

The original contributions presented in the study are included in the article/Supplementary material, further inquiries can be directed to the corresponding author.

## Author contributions

IP: Writing – original draft. E-NT: Writing – original draft. JL: Supervision, Writing – review & editing. US: Supervision, Writing – review & editing. HE-N: Supervision, Writing – review & editing.
