# Supplementary material for: The impact of intermittent fasting on gut microbiota: a systematic review of human studies
Source: Front Nutr. 2024 Feb 12;11:1342787. doi: 10.3389/fnut.2024.1342787 (PMC10894978; doi:10.3389/fnut.2024.1342787)
Supplement: Supplementary file 1 [file Table_1.docx]

**Supplementary Table 1a. Summary of the changes in gut microbiota composition that were observed in fasting groups before vs after IF.** The microbiota composition of each fasting group was measured before and after IF, and comparisons between the two time points were made by using 1. a statistical test which uses p-value or false discovery rate (FDR) as an indicator of statistical significance and/or 2. by using linear discriminant analysis (LDA) or LDA coupled with effect size measurements (LEfSe) analysis which use a specific LDA-score threshold to identify the most differentially abundant taxa at the two time points. In cases where one study/paper contained multiple separate fasting groups or cohorts, the fasting group/cohort is specified after the study reference. There are two such studies in this review: the study by Su et al which had a separate young cohort and a middle-aged cohort, and the study by Ali et al which had two separate fasting groups (Chinese and Pakistani). Regarding the study by Ali et al, besides analyzing the two ethnic groups separately, they also made before vs after IF comparisons including all the participants (Chinese+Pakistani). The meanings of the used symbols are the following: ✢ FDR < 0.1; # LDA-score > 3.00; ## LDA-score > 4.00; ¤ LDA-score not mentioned; * p < 0.05; ** p < 0.01; *** p < 0.005; **** p < 0.001. If there are two different symbols separated by a comma following one study’s reference, it means that the same result was observed in two different analyses within the same study, e.g., from a statistical test using a p-value/FDR and from an LDA/LEfSe-analysis using an LDA-score.

| **Taxa level** | **Bacteria increased in 3 ≥ fasting groups** | **Bacteria increased in 2 fasting groups** | **Bacteria increased in 1 fasting group** | **Bacteria decreased in 1 fasting group** | **Bacteria decreased in 2 fasting groups** | **Bacteria decreased in 3 ≥ fasting groups** |
| --- | --- | --- | --- | --- | --- | --- |
| Phylum | Proteobacteria (Ali Chinese**,#, Ali Chinese+Pakistani**,#, Su young cohort*,##) | Bacteroidetes (Ali Pakistani*,#, Ozkul*) | Spirochaetes✢ (Guo)  Firmicutes*,## (Su young cohort) | Actinobacteria# (Ali Pakistani)  Proteobacteria¤ (Ozkul)  Elusimicrobia¤ (Ozkul)  Chloroflexi# (Guo)  Acidobacteria# (Guo) | Bacteroidetes (Ali Chinese*,#, Su young cohort*,##)    Firmicutes (Ali Pakistani**,#, Ozkul¤) |  |
| Class | Gammaproteobacteria (Ali Chinese#, Ali Chinese+Pakistani#, Su young cohort*,##) | Clostridia (Ali Chinese#, Su young cohort*,##) | Coriobacteriia# (Guo)  Bacilli# (Ali Chinese)  Bacteroidia# (Ali Pakistani)  Negativicutes*,## (Su young cohort) | Bacilli# (Ali Pakistani)  Actinobacteria# (Ali Pakistani)  Mollicutes¤ (Ozkul)  Elusimicrobia¤ (Ozkul)  Thermoleophilia# (Guo)  Ktedonobacteria# (Guo)  Acidobacteriia# (Guo) | Bacteroidia (Su young cohort*,##, Ali Chinese#) | Negativicutes (Su middle-aged cohort*, Ali Pakistani#, Ali Chinese+Pakistani#) |
| Order | Clostridiales (Ali Chinese#, Su young cohort*,##, Su middle-aged cohort***) | Enterobacteriales (Ali Chinese#, Su young cohort*,##) | Pseudomonadales# (Guo)  Coriobacteriales# (Guo)  Lactobacillales# (Ali Chinese)  Bacteroidales# (Ali Pakistani)  Selenomonadales*,## (Su young cohort) | Pasteurellales# (Ali Pakistani)  Coriobacteriales# (Ali Pakistani)  Clostridiales¤ (Ozkul)  Elusimicrobiales¤ (Ozkul)  Solibacterales# (Guo)  Ktedonobacterales# (Guo)  Acidobacteriales# (Guo)  Frankiales# (Guo)  unidentified Alphaproteobacteria# (Guo) | Bacteroidales (Su young cohort*,##, Ali Chinese#)    Lactobacillales (Ali Pakistani#, Ozkul¤)    Enterobacteriales (Ozkul¤, Guo#) | Selenomonadales (Su middle-aged cohort*, Ali Pakistani#, Ali Chinese+Pakistani#) |
| Family |  | *Ruminococcaceae* (Guo#, Su young cohort*,##)    *Moraxellaceae* (Guo#, Ali Chinese#)    *Enterobacteriaceae* (Ali Chinese#, Su young cohort*,##) | *unidentified Flavobacteriales*# (Guo)  *Leuconostocaceae*# (Ali Chinese)  *Streptococcaceae*# (Ali Chinese)  *Clostridiaceae 1*# (Ali Chinese)  *Rikenellaceae*# (Ali Pakistani)  *Porphyromonadaceae*# (Ali Pakistani)  *Lachnospiraceae** (Su middle-aged cohort) | *unidentified_Clostridiales** (Su middle-aged cohort)  *Bacillaceae 1*# (Ali Chinese)  *Pasteurellaceae*# (Ali Pakistani)  *Streptococcaceae*# (Ali Pakistani)  *Coriobacteriaceae*# (Ali Pakistani)  *Elusimicrobiaceae*¤ (Ozkul)  *unidentified Solibacterales*# (Guo)  *Ktedonobacteraceae*# (Guo)  *Corynebacteriaceae*# (Guo)  *Acidothermaceae*# (Guo)  *unidentified Alphaproteobacterial*# (Guo)  *Xanthobacteraceae*# (Guo)  *Prevotellaceae**,## (Su young cohort) | *Lactobacillaceae* (Ali Pakistani#, Ali Chinese+Pakistani#)    *Enterobacteriaceae* (Ozkul¤, Guo#) | *Veillonellaceae* (Su middle-aged cohort*, Ali Pakistani#, Ali Chinese+Pakistani#) |
| Genus | *Faecalibacterium* (Ali Chinese*, Su young cohort*,##, Ozkul¤) | *Roseburia* (Guo#, Ozkul*,¤)    *Butyricococcus* (Guo#, Ozkul¤)    *Klebsiella* (Ali Chinese*,#, Ali Chinese+Pakistani#)    *Enterobacter* (Ali Chinese#, Ali Chinese+Pakistani#)    *Citrobacter* (Ali Chinese#, Ali Chinese+Pakistani#) | *Moraxella*# (Guo)  *Lactococcus*# (Guo)  *Candidatus Soleaferrea*# (Guo)  *Sutterella** (Ali Pakistani)  *Acinetobacter*# (Ali Chinese)  *Weissella*# (Ali Chinese)  *Clostridium XIVb*# (Ali Chinese)  *Anaerotruncus.s uncultured organism*# (Ali Chinese)  *Streptococcus*# (Ali Chinese)  *Corynebacterium*# (Ali Chinese)  *Cronobacter*# (Ali Chinese)  *Clostridium sensu stricto*# (Ali Chinese)  *Escherichia shigella*# (Ali Chinese)  *Anaerobacterium.s uncultured bacterium*# (Ali Pakistani)  *Agathobacter** (Su middle-aged cohort)  *Blautia** (Su middle-aged cohort)  *unidentified_Lachnospiraceae** (Su middle-aged cohort)  *Dialister*¤ (Ozkul)  *Erysipelotrichi*¤ (Ozkul)  *Allobaculum*¤ (Ozkul)  *Akkermansia*¤ (Ozkul)  *Roseburia.s uncultured organism*# (Ali Chinese)  *Dorea***,# (Ali Chinese)  *Dorea.s uncultured organism*# (Ali Chinese)  *Parabacteroides**,# (Ali Pakistani)  *Alistipes**,# (Ali Pakistani)  *Gemmiger.s uncultured organism*# (Ali Chinese)  *Gemmiger*# (Ali Chinese)  *Subdoligranulum**,## (Su young cohort)  *Eubacterium_coprostanoligenes_group**,## (Su young cohort)  *Eubacterium*¤ (Ozkul)  *Bacteroides**,¤ (Ozkul) | *Blautia*** (Ali Pakistani)  *Eubacterium*** (Ali Pakistani)  *Clostridium_XlVa** (Ali Chinese+Pakistani)  *Lachnospiracea incertae sedis** (Ali Chinese+Pakistani)  *Megamonas** (Su middle-aged cohort)  *unidentified_Clostridiales** (Su middle-aged cohort)  *Christensenella.s uncultured organism*# (Ali Chinese)  *Faecalibacterium*# (Ali Pakistani)  *Faecalibacterium.s uncultured organism*# (Ali Pakistani)  *Eubacterium.s uncultured bacterium*# (Ali Pakistani)  *Haemophilus*# (Ali Pakistani)  *Haemophilus.s uncultured organism*# (Ali Pakistani)  *Veillonella*# (Ali Pakistani)  *Veillonella.s uncultured organism*# (Ali Pakistani)  *Collinsella*# (Ali Pakistani)  *Anaerotruncus.s uncultured organism*# (Ali Pakistani)  *Stomatobaculum*# (Ali Chinese+Pakistani)  *Escherichia*¤ (Ozkul)  *Enterococcus*¤ (Ozkul)  *Senegalimassilia*# (Guo)  *Aggregatibacter*# (Guo)  *unidentified Corynebacteriaceae*# (Guo)  *Caproiciproducens*# (Guo)  *Acidothermus*# (Guo)  *Streptococcus**,# (Ali Pakistani)  *Romboutsia**,# (Ali Pakistani)  *Dialister**,# (Ali Pakistani)  *Prevotella 9**,## (Su young cohort) | *Coprococcus* (Ali Pakistani****,#, Ali Chinese+Pakistani**,#)    *Anoxybacillus* (Ali Chinese#, Ali Chinese+Pakistani#)    *Lactobacillus* (Ali Pakistani#, Ali Chinese+Pakistani#) |  |
| Species |  | *Acinetobacter nosocomialis* (Ali Chinese#, Ali Chinese+Pakistani#) | *Ruminococcus bicirculans*# (Guo)  *Clostridium leptum*# (Guo)  *Pasteurella aerogenes*# (Guo)  *Bifidobacterium animalis*# (Guo)  *Lactobacillus amylovorus*# (Guo)  *Ruminococcus gnavus*✢ (Guo)  *Chitinophagaceae bacterium*✢ (Guo)  *Paraburkholderia caribensis*✢ (Guo)  *Verrucomicrobiae bacterium Ellin516*✢ (Guo)  *Neisseria dentiae*✢ (Guo)  *Streptococcus ferus*✢ (Guo)  *uncultured bacterium*# (Ali Chinese)  *Streptococcus salivarius*# (Ali Chinese)  *Prevotellaceae bacterium DJF VR15*# (Ali Chinese)  *Cronobacter dublinensis*# (Ali Chinese)  *Clostridium celatum*# (Ali Chinese)  *Escherichia coli K 12*# (Ali Chinese)  *bacterium mpn solate group 3*# (Ali Pakistani)  *Corynebacterium variabile DSM 44702*# (Ali Chinese+Pakistani)  *Butyricicoccus pullicaecorum**** (Ozkul)  *Faecalibacterium prausnitzii**** (Ozkul)  *Akkermansia muciniphila**** (Ozkul)  *Roseburia faecis*#,✢ (Guo) | *Gamma proteobacterium_SCGC_AAA076_P13*✢ (Guo)  *Acidobacteria bacterium SCN_69_37*✢ (Guo)  *Jatrophihabitans sp*✢ (Guo)  *Gamma proteobacterium W109_152*✢ (Guo)  *Tepidimonas fonticaldi*✢ (Guo)  *Clostridium bornimense*✢ (Guo)  *Brevibacillus thermoruber*✢ (Guo)  *Devosia riboflavina*✢ (Guo)  *Paenibacillus campinasensis*✢ (Guo)  *TM7 phylum sp canine oral taxon 237*✢ (Guo)  *Deltaproteobacteria bacterium_RIFCSPLOWO2_02_56_12*✢ (Guo)  *Paenibacillus aestuarii*✢ (Guo)  *Mitsuokella jalaludinii*✢ (Guo)  *Acidobacteria bacterium WWH111*✢ (Guo)  *Eubacterium sp 1_3*✢ (Guo)  *Christensenellaceae bacterium YE57*✢ (Guo)  *Clostridium_disporicum** (Su middle-aged cohort)  *Victivallis vadensis*# (Ali Chinese)  *uncultured Ruminococcaceae bacterium*# (Ali Chinese)  *uncultured Firmicutes bacterium*# (Ali Pakistani)  *Streptococcus salivarius*# (Ali Pakistani)  *uncultured Clostridiaceae bacterium*# (Ali Pakistani)  *Prevotella disiens*# (Ali Pakistani)  *uncultured Eubacterium sp*# (Ali Chinese+Pakistani)  *Eubacterium rectale ATCC 33656*# (Ali Chinese+Pakistani)  *Oribacterium sp CM12*# (Guo)  *Clostridium innocuum*# (Guo)  *Alistipes indistinctus*# (Guo)  *Lactobacillus salivarius*# (Guo)  *Ruminococcus gnavus*# (Guo) | *Anoxybacillus flavithermus* (Ali Chinese#, Ali Chinese+Pakistani#) |  |

**Supplementary Table 1b. Summary of the changes in gut microbiota composition that were observed in non-fasting control groups before vs after intervention period.** The microbiota composition of each control group was measured before and after intervention period, and comparisons between the two time points were made by using linear discriminant analysis (LDA) which uses a specific LDA-score threshold to identify the most differentially abundant taxa at the two time points. The meanings of the used symbols are the following: # LDA-score > 3.00.

| **Taxa level** | **Increased in 1 paper** | **Decreased in 1 paper** |
| --- | --- | --- |
| Phylum |  |  |
| Class | unidentified Actinobacteria# (Guo) | Thermoleophilia# (Guo) |
| Order | Bifidobacteriales# (Guo) | Gaiellales# (Guo) |
| Family | *Bifidobacteriaceae*# (Guo) | *unidentified Rhizobiales*# (Guo) |
| Genus | *Bifidobacterium*# (Guo)  *Erysipelatoclostridium*# (Guo) | *Candidatus Solibacter*# (Guo)  *Devosia*# (Guo)  *unidentified Burkholderiaceae*# (Guo)  *Veillonella*# (Guo)  *Phocea*# (Guo)  *Catenibacterium*# (Guo)  *Klebsiella*# (Guo) |
| Species | *Actinomyces odontolyticus*# (Guo)  *Erysipelatoclostridium ramosum*# (Guo) | *Coprobacter secundus*# (Guo)  *Klebsiella variicola*# (Guo)  *Burkholderiales bacterium YL45*# (Guo) |

**Supplementary Table 1c. Summary of the differences in gut microbiota composition that were observed between fasting and control groups after intervention period.** The microbiota composition of each group was measured after trial, and comparisons between the two groups were made by using 1. two-tailed t-test which uses a p-value as an indicator of statistical significance or 2. by using linear discriminant analysis (LDA) which uses a specific LDA-score threshold to identify the most differentially abundant taxa between the two groups. The meanings of the used symbols are the following: # LDA-score > 2.5 and p < 0.05; * p < 0.05; ** p < 0.01; *** p < 0.001.

| **Taxa level** | **Higher in fasting group in 2 papers** | **Higher in fasting group in 1 paper** | **Higher in control group in 1 paper** | **Higher in control group in 2 papers** |
| --- | --- | --- | --- | --- |
| Phylum |  | Bacteroidetes# (Zeb healthy males) | Proteobacteria# (Zeb healthy males) |  |
| Class |  | Bacteroidia# (Zeb healthy males) |  |  |
| Order |  | Bacteroidales# (Zeb healthy males) | Enterobacteriales# (Zeb healthy males)  Actinomycetales# (Zeb healthy males) |  |
| Family |  | *Prevotellaceae*# (Zeb healthy males)  *Bacteroidales S24 7 group*# (Zeb healthy males)    *Rikenellaceae*# (Zeb healthy males)  *Clostridiales vadinBB60 group*# (Zeb healthy males) | *Bacteroidaceae*# (Zeb healthy males),  *Enterobacteriaceae*# (Zeb healthy males)  *Actinomycetaceae*# (Zeb healthy males)  *Carnobacteriaceae*# (Zeb healthy males) |  |
| Genus | *Prevotella 9* (Zeb healthy males#, Zeb nutrient intake***)    *Prevotella 2* (Zeb healthy males#, Zeb nutrient intake***)    *Alloprevotella* (Zeb healthy males#, Zeb nutrient intake**)    *Dialister* (Zeb healthy males#, Zeb nutrient intake*)    *Ruminococcaceae UCG 002* (Zeb healthy males#, Zeb nutrient intake*)    *Eubacterium coprostanoligenes group* (Zeb healthy males#, Zeb nutrient intake*)    *Lachnospiraceae NK4A136 group* (Zeb healthy males#, Zeb nutrient intake*)    *Ruminococcus 1* (Zeb healthy males#, Zeb nutrient intake*)    *norank f Bacteroidales S24 7 group* (Zeb healthy males#, Zeb nutrient intake*)    *Coprococcus 2* (Zeb healthy males#, Zeb nutrient intake*)    *Mitsuokella* (Zeb healthy males#, Zeb nutrient intake*) | *Erysipelotrichaceae UCG 004*# (Zeb healthy males)  *unclassified f Veillonellaceae*# (Zeb healthy males)  *Raoultella*# (Zeb healthy males)  *norank f Prevotellaceae*# (Zeb healthy males)  *Paraprevotella*# (Zeb healthy males)  *Slackia*# (Zeb healthy males)  *Howardella*# (Zeb healthy males)  *Oscillibacter*# (Zeb healthy males)  *norank f Clostridiales vadinBB60 group*# (Zeb healthy males)  *norank f Coriobacteriaceae*# (Zeb healthy males)  *Coprococcus 3*# (Zeb healthy males)  *Ruminococcaceae UCG 0T0*# (Zeb healthy males)  *Allisonella*# (Zeb healthy males)  *Ruminococcaceae UCG 005*# (Zeb healthy males)  *Olsenella*# (Zeb healthy males)  *Ruminococcaceae NK4A214 group*# (Zeb healthy males) | *Peptostreptococcus*# (Zeb healthy males)  *Actinomyces*# (Zeb healthy males)  *Granulicatella*# (Zeb healthy males)  *Clostridium innocuum group*# (Zeb healthy males)  *Erysipelatoclostridium*# (Zeb healthy males)  *Elavonitractor*# (Zeb healthy males)  *Eggerthella*# (Zeb healthy males) | *Bacteroides* (Zeb healthy males#, Zeb nutrient intake**)    *Escherichia Shigella* (Zeb healthy males#, Zeb nutrient intake**)    *Ruminococcus gnavus group* (Zeb healthy males#, Zeb nutrient intake*)    *Erysipelotrichaceae UCG 003* (Zeb healthy males#, Zeb nutrient intake*) |
| Species |  |  |  |  |

**Supplementary Table 2. Intermittent fasting (IF) induced changes in weight and/or BMI and changes in energy, macronutrient and food/food group intakes.**

| **Study reference** | **Type of intermittent fasting** | **Method(s) for assessing diet, measurement time points** | **Change in weight and/or BMI** | **Information on energy intake** | **Information on macronutrient intake** | **Information on food/food group intake** |
| --- | --- | --- | --- | --- | --- | --- |
| Guo et al., 2021 | 5:2 diet | A 3-day (2 workdays and 1 off-day) dietary recall interview at baseline and after 8-weeks of 5:2 diet.    5:2 diet group were asked to record what they ate and the cooking methods they used on every fasting day and to take pictures of the food in real time. | In the 5:2 diet group, BMI was significantly reduced from 29.3 kg/m^2^ (95% CI, 27.4-31.2) to 28.0 kg/m^2^ (95% CI, 26.2-29.8), and weight decreased from 77.8 kg (95% CI, 71.6-84.0) to 74.3 kg (95% CI, 68.4-80.2) (both p<0.001) between baseline and 8 weeks. There was no significant change in the control group. | There was no significant difference in baseline calorie intakes between the 5:2 diet and control group. In the process of the trial, participants in the 5:2 diet group had significantly reduced daily calorie intake on fasting days, averaging 31.0% (95% CI, 27.8%- 34.2%) of the intake on non-fasting days. On non-fasting days, calorie intake was maintained compared to baseline, with no significant increase in the 5:2 diet group. Participants in the control group did not differentially modify their energy intakes during the trial. | There was no significant difference in baseline macronutrient intakes between the 5:2 diet and control group.  Participants in the control group did not differentially modify their macronutrient intakes during the trial. | Not stated. |
| Cignarella et al., 2018 | ADF | A 7-day food recall diary, seemingly both before and after study period.  Only those subjects randomized to the ADF group were instructed on the ADF diet by the study dietician. | The ADF and control group had no significant difference in BMI at baseline (ADF 30.2 ± 5.8 kg/m^2^ vs. control 31.2 ± 6.4 kg/m^2^; p=0.7). However, there was a significant difference in BMIs on day 15 in the two groups after controlling for baseline BMI (ADF 29.1 ± 5.3 kg/m^2^ vs. control 31.1 ± 6.5 kg/m^2^; p=0.03 by ANCOVA). Values are expressed as mean ± SD. | During the fasting days, the ADF group was allowed to eat one or two salads (fresh or steamed non starchy vegetables) with a light dressing, not to go over 500 calories/day. No further information was reported of the ADF group’s energy or macronutrient intakes on non-fasting days or those of the non-fasting control group. | Not stated. | Not stated. |
| Zeb et al., 2020. Healthy males | TRF | It seems that dietary intake was measured only at one time point, and it is unclear when this was. The method used for measuring dietary intake was not stated either. | BMI and weight of the TRF and control group were reported only at one time point, and it was unclear whether this was before or after the intervention period. At this time point, BMI of the TRF group (24.14±3.5 kg/m^2^) was significantly lower than that of the control group (26.13±5.2 kg/m^2^) (p=0.046). There was no statistically significant difference between the two groups in body weight. Values reported as mean ± SD. | At the measured time point, the TRF group had significantly (p<0.05) smaller intakes of energy (kcal/day) than the control group. | At the measured time point, the TRF group had significantly (p<0.05) smaller intakes (g/day) of protein, fat and carbohydrate than the control group. There were no significant differences in water and dietary fiber intake. | Not stated. |
| Gabel et al., 2020 | TRF | Energy intake was assessed by a 7-day food record during the baseline period and at W12. | Body weight was significantly reduced (p<0.05) after 12 weeks of TRF (B1: 94.85 ± 3.77; W12: 93.23 ± 4.23; change: -2.05 ± 0.75 0.017). Values reported as mean ± SEM. | Energy intake was reduced (p<0.05) from 1832 ± 153 kcal/day at B1 to 1461± 114 kcal/day at W12 (-371 ± 136 kcal/d = 20% overall energy restriction). | Macronutrient intakes were reported in Gabel et al 2018 as % of total energy: 16±1 protein, 47±2 carbohydrate, 37±1 fat at B1, and 17±1 protein, 46±2 carbohydrate, 37±2 fat at W12. Values reported as mean ± SEM. | Not stated. |
| Ozkul et al., 2020 | TRF | Dietary intake was assessed only before the intervention with a short dietary questionnaire, which was used to determine habitual dietary intake and exclude high fat and/or high glucose food consumption. | All participants were within the normal range in terms of BMI (23.0±1.5 kg/m^2^) at baseline. No information about the participants’ BMI or weight was reported after the intervention. | Not stated. | Not stated. | Not stated. |
| Ali et al., 2021 | TRF | Dietary information was collected before and after Ramadan fasting. A 3 day 24-h food dietary recall was defined for the consumption of total foods and beverages. This questionnaire was also used to assess the dietary record of the last 6 months for each individual. | No information about the participants’ weight or BMI was reported neither before nor after the intervention period. | CBF vs CAF: no significant differences in energy intake (kcal/day)  PBF vs PAF: no significant differences in energy intake (kcal/day) | CBF vs CAF: no statistically significant differences in daily carbohydrate, protein or fat intakes (%energy)  PBF vs PAF: no statistically significant differences in daily carbohydrate, protein or fat intakes (%energy) | CBF vs CAF: significant differences in several food group intakes (g/day)  PBF vs PAF: significant differences in several food group intakes (g/day) |
| Zeb et al., 2020. Nutrient intake | TRF | A 3-d food diary was recorded by each student following study protocol. | No information about the participants’ BMI or weight was reported neither before nor after the intervention period. | The TRF group had significantly (p<0.05) smaller intakes of energy (kcal/day) than the control group. | The TRF group had significantly (p<0.05) smaller intakes (g/day) of protein, fat and carbohydrate than the control group. There were no significant differences in water and dietary fiber intake. | Not stated. |
| Su at al., 2021 | This article consisted of two separate cohorts, which are addressed separately below. |  |  |  |  |  |
| Su at al., 2021. Young male adult cohort | TRF | Information on food intake was not collected. | 30 days of TRF caused a statistically significant loss of 3.45% in body weight (p<0.001). | Not collected. | Not collected. | Not collected. |
| Su at al., 2021. Middle- aged cohort | TRF | Modified FFQ to assess dietary intake 1 month before fasting, during fasting, and a month after the cessation of the fasting. | TRF group: statistically significant differences in body weight between T1 vs T2 (63.62 ± 9.89 vs. 62.47 ± 9.65; p<0.001) and T1 vs T3 (63.62 ± 9.89 vs. 62.78 ± 9.48; p=0.005), but not between T2 vs T3 (62.47 ± 9.65 vs. 62.78 ± 9.48; p=0.11), indicating that weight decreased during Ramadan fasting and remained relatively same during the 30-day follow-up period. In control group, there were no statistically significant differences in body weight between different time points. | In the TRF group energy intake (kcal/week) was significantly different between T1 vs T2 and T2 vs T3 (p<0.001 for both), but not between T1 and T3, indicating that the calorie intake was significantly reduced during Ramadan and returned to baseline levels after it was over. In the control group, there were no statistically significant differences in energy intake (kcal/week) between different time points. Between the two groups, there was a significant difference only at T2 (p=0.03). | Not stated. | Not stated. |

Abbreviations in alphabetical order: ADF=alternate day fasting, ANCOVA=analysis of covariance, B1=beginning of the baseline period, BMI=body mass index, CAF=Chinese after fasting, CBF=Chinese before fasting, FFQ= food frequency questionnaire, PAF=Pakistani after fasting, PBF=Pakistani before fasting, T1=day 0 (start of the 2018 Ramadan), T2=day 30 (end of the 2018 Ramadan), T3=day 60 (1 month after the end of Ramadan fasting), TRF=time-restricted eating, W12=after 12 weeks of TRF
